# The Impact of Total Tumor Diameter on Lymph Node Metastasis and Tumor Recurrence in Papillary Thyroid Carcinomas

**DOI:** 10.3390/diagnostics14030272

**Published:** 2024-01-26

**Authors:** Nuray Can, Buket Yilmaz Bulbul, Filiz Ozyilmaz, Necdet Sut, Meltem Ayyıldız Mercan, Burak Andaç, Mehmet Celik, Ebru Tastekin, Sibel Guldiken, Yavuz Atakan Sezer, Semra Ayturk Salt, Ezgi Genç Erdoğan, Funda Ustun, Hakan Gurkan

**Affiliations:** 1 Department of Pathology, Faculty of Medicine, Trakya University, 22030 Edirne, Türkiye; fozyilmaz@yahoo.com (F.O.); ayyildizmltm@gmail.com (M.A.M.); ebrutastekin@hotmail.com (E.T.); ezgigencc@gmail.com (E.G.E.); 2 Department of Internal Medicine, Division of Endocrinology and Metabolism, Faculty of Medicine, Trakya University, 22030 Edirne, Türkiye; buketylmz@hotmail.com (B.Y.B.); burakandac@trakya.edu.tr (B.A.); drmehmetcelik@hotmail.com (M.C.); sibel71@hotmail.com (S.G.); 3 Department of Biostatistics, Faculty of Medicine, Trakya University, 22030 Edirne, Türkiye; nsut@trakya.edu.tr; 4 Department of General Surgery, Faculty of Medicine, Trakya University, 22030 Edirne, Türkiye; atakansezer@hotmail.com; 5 Department of Internal Medicine, Division of Endocrinology and Metabolism, Kayseri City Hospital, 38080 Kayseri, Türkiye; s.ayturk@hotmail.com; 6 Department of Nuclear Medicine, Faculty of Medicine, Trakya University, 22030 Edirne, Türkiye; fundaustun@trakya.edu.tr; 7 Department of Medical Genetics, Faculty of Medicine, Trakya University, 22030 Edirne, Türkiye; dr_hakangurkan@yahoo.de

**Keywords:** papillary thyroid carcinoma, multifocality, bilaterality, total tumor diameter, number of tumor foci, perineural invasion

## Abstract

(1) Introduction: The impact of multifocality/bilaterality on the prognosis of papillary thyroid carcinoma (PTC) is a matter of debate. In order to clarify this debate, several studies have attempted to identify additional parameters associated with poor prognosis, including total tumor diameter (TTD), in the context of multifocal PTCs. In this context, this study was carried out to investigate the impact of TTD on tumor recurrence and lymph node metastasis (LNM) in PTCs. (2) Materials and Methods: The sample of this single-center retrospective study consisted of 706 patients diagnosed with PTC. TTD was calculated as the sum of the largest diameters of tumor foci in multifocal tumors. The resulting TTDs were grouped into TTDs ≤ 10 mm, TTDs > 10 mm, TTDs ≤ 20 mm, and TTDs > 20 mm, using 10 mm and 20 mm as cutoff values. (3) Results: There was no significant difference between multifocal papillary microcarcinomas (PTMCs) with a TTD of >10 mm and unifocal PTCs with a primary tumor diameter (PTD) of >10 mm except for advanced age and lymphovascular invasion (LVI). In addition, perineural invasion (PNI) and TTD > 10 mm were found to be significant risk factors for LNM, and PNI, TTD > 10 mm, TTD > 20 mm, and bilaterality were found to be significant risk factors for recurrence. LVI, and TTD > 10 mm were found to be independent significant predictors for recurrence, and LVI and extrathyroidal extension (ETE) were found to be independent significant predictors for LNM. (4) Conclusions: Considering TTD > 10 mm in recurrence risk categorization models and adopting a clinical approach that takes into account multifocal PTMCs with TTD > 10 mm along with unifocal PTCs with PTD > 10 mm may be more useful in terms of clinical management of the disease.

## 1. Introduction

The prevalence of thyroid carcinoma, the most common neoplasm of the endocrine glands, has tripled since 1975. Despite the epidemic of thyroid cancer diagnosis, mortality rates associated with thyroid cancer have remained stable due to favorable prognoses [1,2,3]. Then again, some of the thyroid carcinomas may present in a clinically aggressive manner. To this end, staging systems, including the most recent eighth edition of the staging system designed by the American Joint Committee on Cancer (AJCC) [4,5,6], have defined some parameters associated with aggressive tumor behavior. The relevant clinical management guidelines adopted by the American Thyroid Association (ATA) [4], the American Association of Clinical Endocrinologists [7], The British Thyroid Association [8], and the National Cancer Center Network (NCCN) [9] have been based on these parameters.

Multifocality and bilaterality are common features of PTCs and PTMCs, with prevalence rates ranging from 18% to 87% and 13% to 46%, respectively [10,11,12,13,14,15]. Although the ATA [4] and NCCN [9] current management guidelines consider multifocality, among other risk factors, in risk scoring for thyroid carcinoma, neither current staging systems [6] nor current clinical management guidelines consider multifocality or bilaterality as stand-alone parameters in pathological staging or risk categorization of PTCs [4,9]. Although several recent studies [16,17,18,19,20,21,22] have reported the adverse effects of multifocality and/or bilaterality on the prognosis of PTC, global data on the relationship between multifocality and bilaterality and the clinicopathological features of PTC remain controversial [10,16,17,18,23,24,25,26,27,28,29,30]. The multifocality of PTC usually presents with incidental tumors that are smaller in size than the primary tumor that constitutes one of the general targets of surgery [24]. However, it is not clear whether these unevaluated tumors pose a higher risk [25], especially in patients undergoing hemithyroidectomy [31]. To this end, several studies have attempted to identify additional parameters associated with poor prognosis, including total tumor diameter (TTD), total surface area of the tumor (TSA), tumor diameter ratio (TDR), and number of tumor foci (TN), in the context of PTCs [17,18,24,25,32,33,34,35]. Most of these studies reported higher extrathyroidal extension (ETE) and lymph node metastasis (LNM) rates in PTCs with a TTD of >10 mm than in PTCs with a TTD of ≤10 mm [18,26]. One of these studies reported that patients with multifocal PTMCs with a TTD of >10 mm had higher rates of ETE, LNM, and capsular invasion than those with unifocal PTMCs. In addition, they stated that they did not find any significant difference between patients with multifocal PTMCs with a TTD of >10 mm and those with unifocal PTCs with a PTD of >10 mm in any of the parameters they investigated [18]. However, it was also reported that, unlike the TSA value, which significantly predicted LNM, a TTD cutoff value of >10 mm did not significantly predict LNM [25]. Although these studies demonstrated the predictive values of these parameters, they did not reveal their usability in relevant staging systems and clinical management guidelines.

In view of the foregoing, this study was carried out to investigate the impact of TTD on tumor recurrence and lymph node metastasis along with focality, laterality, and TN in a PTC cohort with a mean duration of follow-up of approximately 10 years, based on a review of the literature on the effects of TTD in PTCs.

## 2. Materials and Methods

### 2.1. Study Design

**Figure 1:**
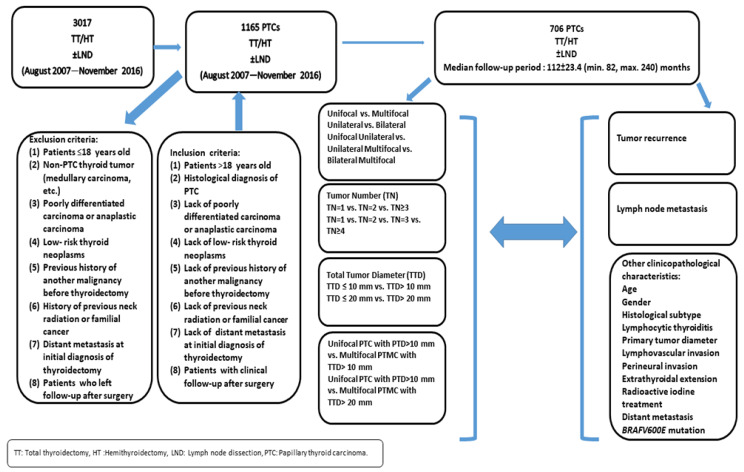
Study design.

This study was designed as a single-center retrospective study. The study protocol was approved by the local Ethics Committee of the University Hospital. Medical records and histopathological reports of 3017 patients who had undergone total thyroidectomy/hemithyroidectomy with or without central and/or lateral cervical lymph node dissection in the Department of Pathology were reviewed retrospectively (between August 2007 and November 2016). The study design is summarized in Figure 1.

Medical records and histopathological reports of 1165 patients with the diagnosis of PTC were evaluated. Patients’ medical records and histopathological reports were reviewed, and 459 patients were excluded from the study according to the inclusion and exclusion criteria, which are presented in Figure 1. Finally, 706 patients (589 (83.4%) females and 117 (16.6%) males) were enrolled into the study.

### 2.2. Histopathological Features

Patients’ histopathological features were obtained from the pathology records of thyroidectomy materials without microscopic reevaluation. At the initial pathological evaluation, all or almost all of the thyroidectomy materials were sampled. In very large thyroidectomy materials, at least 10 samples per lobe were evaluated, including all macroscopically observed lesions. Histopathological examination was performed by pathologists experienced in thyroid pathology. Histological subtypes of PTC were classified as classic (CPTC), infiltrative follicular (IFPTC), and aggressive variants of (AVPTC) subtype in accordance with the fifth edition of the World Health Organization (WHO) Classification of Thyroid Tumors (Beta version) [36]. Other histopathological features of the patients that were statistically analyzed included tumor-surrounding lymphocytic thyroiditis (LT), primary tumor diameter (PTD) (diameter of the largest tumor foci in multifocal tumors), lymphovascular invasion (LVI), perineural invasion (PNI), lymph node metastasis (LNM), and extrathyroidal extension (ETE). Given that the study data were obtained by retrospectively reviewing the patients’ records and the study group included patients between 2007 and 2016, histopathological features such as minor ETE and gross ETE could not be evaluated.

#### 2.2.1. Focality/Laterality and Number of Tumor Foci

**Figure 2:**
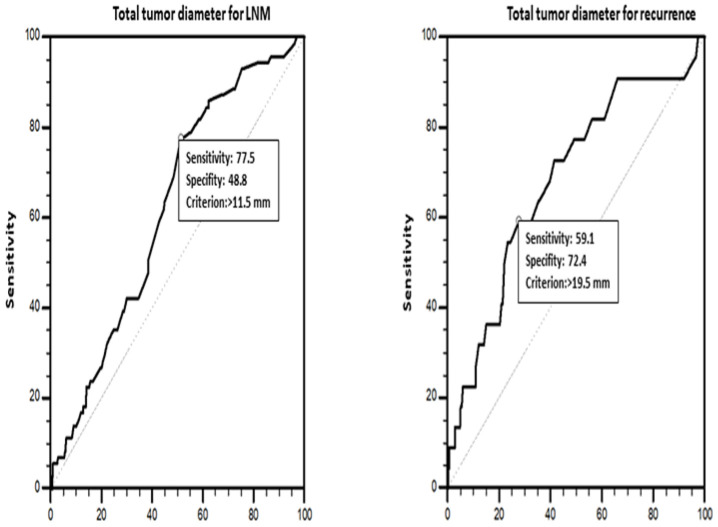
ROC analysis for total tumor diameter affecting lymph node metastasis and recurrence.

Patients with two or more tumor foci were included in the multifocal group. Laterality was evaluated for patients with total thyroidectomy. The presence of tumors in both thyroid lobes was considered to indicate bilaterality. Multifocal tumors located in a single lobe and isthmus of the thyroid were classified as unilateral tumors. Additionally, tumors in patients with total thyroidectomy were classified as unilateral unifocal, unilateral multifocal, or bilateral multifocal tumors. A receiver operating characteristics (ROC) curve analysis (Figure 2) was performed to determine the optimal cutoff value for the number of tumor foci (TN) in predicting LNM and recurrence. Three-tiered (1, 2, and ≥3 foci) and four-tiered (1, 2, 3, and ≥4 foci) systems were used to group TNs. Accordingly, it was determined that patients with multiple tumor foci may be more likely to have LNM and recurrence (AUC = 0.523 (standard error = 0.0350); *p* = 0.511) (sensitivity 59.1%, specificity 46.8%).

#### 2.2.2. Total Tumor Diameter

**Figure 3:**
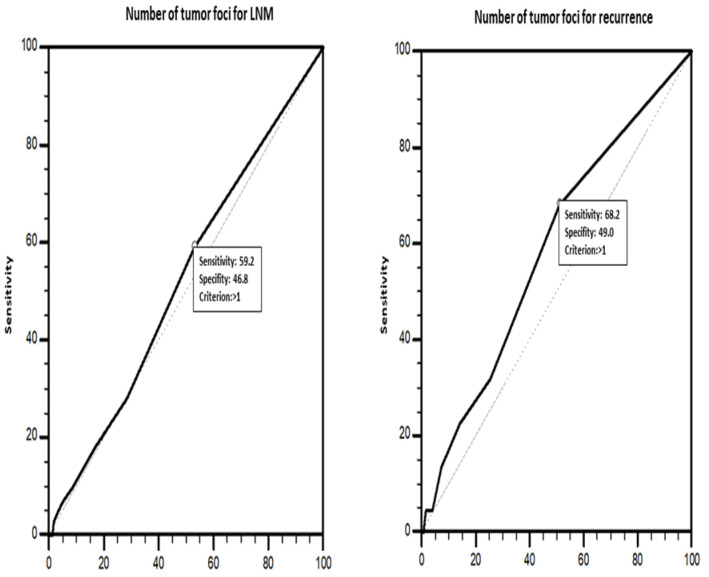
ROC analysis for number of tumor foci affecting lymph node metastasis and recurrence.

TTD was calculated as the sum of the largest diameters of tumor foci in multifocal tumors. The resulting TTDs were grouped into TTDs ≤ 10 mm, TTDs > 10 mm, TTDs ≤ 20 mm, and TTDs > 20 mm, using 10 mm [18] and 20 mm [26] as cutoff values. The clinicopathological characteristics of the patients were compared among these groups. An ROC curve analysis (Figure 3) revealed 11.5 mm (area under the curve (AUC) = 0.621, standard error (SE) = 0.0333; *p* = 0.0003) (sensitivity: 77.5%, specificity: 48.8%, *p* = 0.0003) and 19.5 mm (AUC = 0.682 (SE = 0.0610); *p* = 0.0028) (sensitivity: 59.1%, specificity: 72.4%, *p* = 0.0028) as the optimum cutoff values for TTD in predicting LNM and recurrence. The univariate and multivariate analyses for LNM and recurrence were conducted considering these cutoff values.

### 2.3. Clinical Features and Follow-Up Data

Patients’ clinical features, including whether postoperative radioactive iodine (RAI) therapy was received, presence of distant metastasis, and recurrence, were obtained from the patient information forms available in the hospital information system. The decision on the surgical procedure to be performed was made by the multidisciplinary council based on the applicable ATA guidelines. Surgical procedures were performed by surgeons highly experienced in thyroid surgery. Given the high incidence of lymphocytic thyroiditis and associated peri-thyroidal lymphadenomegaly in our region, excision of the central region lymph nodes was also included among the surgical procedures to be performed if detected during the surgery, regardless of whether the lymph node dissection decision was made before the surgery.

During the follow-up visits, patients underwent a physical examination, thyroid function tests, i.e., free thyroxin, thyroid-stimulating hormone (TSH), thyroglobulin (Tg) and anti-thyroglobulin (Anti-Tg) tests, neck ultrasonography, chest radiography, and whole body iodine scan every 6–12 months for the first 2 years and once a year thereafter. Patients with significantly elevated Tg and/or Tg antibody levels were subjected to additional imaging (computed tomography, magnetic resonance imaging, positron emission tomography/computed tomography) or histological examinations.

RAI therapy has been provided in our center since 2013. The need for RAI therapy and the dose of RAI therapy was planned by the multidisciplinary endocrine diseases council based on the risk stratification of the patients determined according to the histological type, tumor diameter, presence of aggressive histology, presence of LVI, and presence of ETE, in line with the applicable ATA guidelines (Table 5, Recommendation 31 of 2009 ATA guidelines [37], and Table 11, Table 14, Figures 4–8, Recommendations 47 and 48 of 2015 ATA guidelines [4]). Post-treatment I-131 imaging was performed between the seventh and twelfth days after RAI treatment to evaluate whether patients had distant metastases.

Localized, regional, or distant recurrences in patients who initially met remission criteria were considered new histopathological evidence.

### 2.4. Molecular Analysis

The *BRAFV600E* mutation was also included in the statistical analyses since its presence increases the recurrence risk according to the current ATA guidelines [4]. However, since molecular analyses of thyroid cancers started to be performed in our center in 2011, *BRAF* mutation analysis could be performed on only 455 of the 706 patients constituting the study sample. The records of the molecular analysis of the *BRAF* gene were obtained from the Department of Medical Genetics and the Laboratory of Molecular Pathology of the Department of Pathology. In these 455 patients, the tumor tissue containing at least 10–30% of tumor cells was isolated from the sections of the largest tumor foci. Subsequently, DNA purification was performed using the nucleic acid isolation kit for paraffin-embedded tissue (QIAamp^®^ DNA FFPE Tissue Kit (50), QIAGEN (Hilden, Germany) Catalog No. 56404, EZ1^®^ DNA Tissue Kit (48), QIAGEN (Hilden, Germany) 953034, PAXgene^®^ Tissue Containers (10), QIAGEN (Hilden, Germany) Catalog No. 765112, PAXgene Tissue DNA Kit (50), QIAGEN (Hilden, Germany) Catalogue No. 767134). Per the polymerase chain reaction (PCR) procedures, pyrosequencing analysis was performed on PyroMarkQ24, using sequencing primers including the Seq Primer *BRAF 600E* or Seq Primer *BRAF 600E* 464–469 (QIAGEN Catalogue No. 970470).

### 2.5. Statistical Analysis

The results of the statistical analyses were given in mean with standard deviation, median with minimum and maximum, and number and percentage values. The chi-square test (Pearson, Yates, or Fisher) was used in comparing patients’ clinicopathological features according to the tumor laterality and focality (unifocal unilateral/multifocal unilateral/multifocal bilateral), TN, and total tumor diameter (TTD (≤10 mm vs. >10 mm), TTD (≤20 mm vs. >20 mm)). Univariate logistic regression analysis was performed to determine the clinicopathological parameters significantly predicting recurrence and LNM (*p* < 0.05). The parameters found to be significantly predicting recurrence and LNM, i.e., TTD and TN, were further evaluated by multivariate backward stepwise logistic regression analysis by calculating the respective odds ratios (ORs) within a 95% confidence interval (CI). ROC curve analyses were performed to determine the optimum cutoff values of TTD and TN in predicting recurrence and LNM. The AUC, sensitivity, and specificity values of TTD and TN for the given cutoff values were calculated. Probability (*p*) statistics of <0.05 were deemed to indicate statistical significance. The SPSS 20.0 (Statistical Product and Service Solutions for Windows, Version 20.0, IBM Corp., Armonk, NY, USA, 2011) software package was used to conduct the statistical analyses.

## 3. Results

### 3.1. Patients’ Clinicopathological Characteristics

The median age of the sample was 49.55 years. There were 235 (33.3%) patients aged ≤45 years and 471 (66.7%) aged >45 years. Of the 706 patients in the sample, 366 (51.8%) had multifocal tumors. There was a single tumor in 342 (48.4%) patients, two tumor foci in 183 (25.9%) patients, three tumor foci in 79 (11.2%) patients, and four or more tumor foci in 102 (14.5%) patients. Bilateral tumors were present in 228 (34.1%) of 668 patients who underwent total thyroidectomy. In terms of the histological subtype of the tumor, 346 (49%) patients had CPTC, 336 (47.6%) had IFPTC, and 24 (3.4%) had ASPTC. In terms of tumor size, 406 (57.5%) patients had microcarcinoma and 300 (42.5%) patients had a tumor larger than 10 mm. Lymph node metastasis was present in 72 (16.6%) of the 434 patients who underwent lymph node dissection. ETE was present in 140 (19.8%) patients. LVI was observed in 67 (9.5%) patients and PNI in 13 (1.8%) patients. Lymphocytic thyroiditis accompanied PTC in 295 (41.7%) patients. Of the 706 patients, 380 (53.8%) received radioactive iodine therapy, 22 (3.1%) had recurrence, and 1 (0.1%) had distant metastasis. TTD was larger than 10 and 20 mm in 383 (54.2%) and 178 (25.2%) patients, respectively. Of the 455 patients analyzed for the *BRAFV600E* mutation, 93 (20.4%) had it (Table 1). Clinicopathological features of papillary microcarcinomas are also presented in Table 1. The mean duration of follow-up was 112 ± 23.4 (min. 82, max. 240) months.

### 3.2. Distribution of Clinicopathological Features According to Focality and Number of Tumor Foci

The distribution of clinicopathological features by focality revealed that most multifocal tumors featured PTCs larger than 10 mm (*p* < 0.001) and that they were significantly more common in older patients (≥45 years) (*p* = 0.018). The need for RAI therapy was significantly more common in patients with multifocal tumors (*p* < 0.001). The presence of LVI (*p* = 0.023), the presence of ETE (*p* = 0.008), and a higher frequency of RAI therapy (*p* < 0.001) were found to be significantly associated with multifocality in microcarcinomas. There was a near-significant relationship between multifocality and older age (>45 years) (*p* = 0.061), male gender (*p* = 0.067) and LNM (*p* = 0.081 in PTMCs). (Supplementary Table S1).

The analysis of the relationships between TN and clinicopathological features revealed that unifocal tumors were significantly more common in younger (≤45 years) patients (*p* = 0.049 (for four-tiered system) and *p* = 0.031 (for three-tiered system)) and in tumors with a PTD of ≤10 mm (*p* < 0.001) (Supplementary Table S2). There was a significant positive correlation between the rate of patients needing RAI therapy and TN (*p* < 0.001). The ASPTC subtype was significantly more common in tumors with two foci. There was a significant negative correlation between IFPTC and TN (*p* < 0.001 and *p* = 0.006). The comparison of prognostic clinicopathological parameters within the patient subgroup featuring PTMCs revealed significant positive correlations between TN and the rate of LVI (*p* = 0.036 (for four-tiered system) and *p* = 0.032 (for three-tiered system)), the rate of ETE (*p* = 0.045 (for four-tiered system) and *p* = 0.022 (for three-tiered system)), and the need for RAI therapy (*p* < 0.001).

### 3.3. Comparisons of Clinicopathological Features among Unilateral Unifocal PTCs, Unilateral Multifocal PTCs, and Bilateral Multifocal PTCs

Comparisons of clinicopathological features among unilateral unifocal PTCs, unilateral multifocal PTCs, and bilateral multifocal PTCs revealed that, for patients with PTDs larger than 10 mm (*p* < 0.001), RAI therapy (*p* < 0.001) and recurrence (*p* = 0.028) were significantly more common among patients with bilateral multifocal tumors. Significantly more patients with multifocal microcarcinomas, either unilateral or bilateral, needed RAI therapy (*p* = 0.002). The incidence of ETE was lower, albeit not significantly, in patients with unilateral unifocal tumors (*p* = 0.093) (Supplementary Table S3).

### 3.4. Distribution of Clinicopathological Features by Total Tumor Diameter

#### 3.4.1. Comparison of Clinicopathological Features between Patients with a TTD of >10 mm and Patients with a TTD of ≤10 mm

The comparison of the clinicopathological features between patients with a TTD of >10 mm and patients with a TTD of ≤10 mm indicated that male gender (*p* = 0.032), larger tumor size (>10 mm) (*p* < 0.001), LVI (*p* < 0.001), PNI (*p* = 0.008), LNM (*p* < 0.001), ETE (*p* < 0.001), the need for RAI therapy (*p* < 0.001), and *BRAFV600E* mutation (*p* = 0.012) were significantly more common in patients with a TTD of >10 mm than in patients with a TTD of ≤10 mm (Table 2). In addition, recurrence was more common, albeit not significantly, in patients with a TTD of >10 mm than in patients with a TTD of ≤10 mm (*p* = 0.078).

#### 3.4.2. Comparison of Clinicopathological Features between Patients with a TTD of >20 mm and Patients with a TTD of ≤20 mm

The comparison of the clinicopathological features between patients with a TTD of >20 mm and patients with a TTD of ≤20 mm indicated that male gender (*p* = 0.014), larger tumor size (>10 mm) (*p* < 0.001), aggressive histological subtype (*p* = 0.002), LVI (*p* < 0.001), PNI (*p* = 0.017), LNM (*p* = 0.044), ETE (*p* < 0.001), recurrence (*p* = 0.005), and the need for RAI therapy (*p* < 0.001) were significantly more common in patients with a TTD of >20 mm than in patients with a TTD of ≤20 mm (Table 2).

#### 3.4.3. Comparison of Clinicopathological Features between Patients with Unifocal Papillary Thyroid Carcinomas Larger Than 10 mm and Patients with Multifocal Papillary Microcarcinomas with a TTD of >10 mm and >20 mm

The comparison of the clinicopathological features between patients with unifocal tumors with a PTD of >10 mm and multifocal PTMCs with a TTD of >10 mm revealed significant correlations between multifocal PTMCs with a TTD of >10 mm and older age (>45 years) (*p* = 0.011) and between unifocal tumors with a PTD of >10 mm and LVI (*p* = 0.025) and the need for RAI therapy (*p* < 0.001) (Table 3). Additionally, there were significant correlations between multifocal PTMCs with a TTD of >10 mm and older age (>45 years) (*p* = 0.011) and between unifocal tumors with a PTD of >10 mm and LVI (*p* = 0.025) and the need for RAI therapy (*p* < 0.001).

The analysis of the relationship between unifocal tumors with a PTD of >10 mm and multifocal PTMCs with a TTD of >20 mm revealed significant correlations between multifocal PTMCs with a TTD of >20 mm and CPTC (*p* = 0.020) and between unifocal tumors with a PTD of >10 mm and the need for RAI therapy (*p* = 0.005) and ASPTC (*p* = 0.020) (Table 3).

### 3.5. Clinicopathological Features Predictive of LNM

The univariate analysis determined by the ROC curve analysis revealed the presence of LVI (29.640 (14.901–58.956)), presence of PNI (8.285 (2.275–30.170)), presence of ETE (7.170 (4.148–12.395)), male gender (3.903 (2.169–7.023)), TTD > 11.5 mm (performed according to the TTD cutoff value of 11.5 mm in predicting LNM) (3.235 (1.787–5.857)), TTD > 10 mm (3.009 (1.641–5.517)), presence of *BRAFV600E* mutation (2.850 (1.494–5.437)), larger tumor size (>10 mm) (2.554 (1.504–4.338)), older age (≤45 years) (0.537 (0.321–0.899)), and ASPTC (0.261 (0.137–0.494)) as significant predictors for LNM with decreasing OR values (Table 4). Further analyses of these factors by multivariate analysis revealed LVI (10.305 (2.591–40.987)) and ETE (4.608 (1.388–15.302)) as independent significant predictors for LNM (Table 5).

### 3.6. Clinicopathological Features Predictive of Recurrence

The univariate analysis determined by the ROC curve analysis revealed the presence of LVI (7.472 (3.064–18.222)), presence of PNI (6.118 (1.272–29.434)), presence of ETE (4.302 (1.825–10.141)), TTD > 20 mm (3.745 (1.589–8.824)), *BRAFV600E* mutation (3.602 (1.271–10.204)), TTD > 19.5 mm (performed according to the TTD cutoff value of 19.5 mm in predicting recurrence) (3.460 (1.262–9.484)), larger tumor size (>10 mm) (3.000 (1.208–7.452)), TTD > 10 mm (2.954 (1.078–8.098)), and bilaterality (2.406 (1.023–5.656)) as significant predictors for tumor recurrence (Table 4). Further analyses of these factors by multivariate analysis revealed a TTD cutoff value of 10 mm and LVI (12.146 (2.269–65.019)) as independent significant predictors for tumor recurrence (Table 5).

## 4. Discussion

PTC is the most common thyroid carcinoma with a favorable prognosis [1,2,3]. Then again, some of the thyroid carcinomas may present in a clinically aggressive manner, mainly due to localized and distant recurrences. Current guidelines stratify the patient groups at risk for PTC based on parameters that predict the recurrence of tumors, such as tumor size, ETE, LNM, and *BRAF*/*TERT* mutations [4,38]. Nevertheless, decision making regarding the clinical management of PTCs can be challenging, especially considering the still controversial multifocal and bilateral tumors. To this end, several studies have attempted to identify additional parameters associated with poor prognosis, including TTD and TN, in the context of multifocal PTCs [17,18,24,25,32,33,34,35]. In parallel, in this study, a set of currently used parameters, such as *BRAFV600E* mutation, and potential parameters, such as TTD, TN, and tumor focality/laterality, were investigated in patients with PTCs with a median follow-up duration of 9.3 years. The major findings of this study were that (1) multifocality and bilaterality may impact tumor behavior in PTCs, particularly in PTMCs, (2) multifocal PTMCs with a TTD of >10 mm and unifocal PTCs with a PTD of >10 mm may have similar risk factors for tumor recurrence, and TTD may be an effective risk factor for recurrence and LNM in PTCs, and (3) PNI shall not be overlooked, as it is likely that PNI, along with TTD and other risk factors included in recurrence risk categorization, may be neglected as an indicator of LNM and recurrence in PTCs.

Multifocality and bilaterality are common features of PTCs and PTMCs, with prevalence rates ranging from 18.0% to 87.5% [22,39,40,41] and 13.3% to 46.0% [17,32,42,43], respectively. Similarly, in this study, the rate of multifocality was found to be 51.8% in PTCs and 46.1% in PTMCs, whereas the rate of bilaterality was found to be 34.1% in PTCs and 27.7% in PTMCs. Higher rates of bilaterality may be due to the choice of total thyroidectomy as the type of surgical procedure and the fact that histopathological evaluation of all or almost all thyroidectomy materials was performed.

The prognostic effect of multifocality has been debated since the 1960s [26,27,28,41,44,45,46,47,48,49,50,51]. In current clinical management guidelines, multifocality is not considered a stand-alone prognostic factor [4,9]. It has been reported in several large case series that multifocality is not related to recurrence or clinical outcomes in PTCs [10,52,53,54]. On the other hand, some studies found a significant relationship between multifocality and adverse prognostic features, including higher tumor stage [25,28,42,44,55], higher risk for LNM [18,19,25,26,28,43,49,55,56,57,58,59,60,61,62], and higher rate of recurrence [10,27,28,30,44,56,63]. The comparison of unifocal and multifocal groups in this study revealed significant correlations between multifocality and tumor size, older age, and the need for RAI therapy but did not reveal significant correlations between multifocality and recurrence or LNM. LNM was more common in patients with multifocal PTMCs, albeit not significantly. Furthermore, LVI and ETE were observed more commonly in multifocal PTMCs than unifocal PTMCs. Kim et al. [28] reported that patients with multifocal PTCs, particularly PTMCs with tumor foci ≥5, had higher recurrence rates. Qu et al. [56] reported significant positive correlations among adverse prognostic features, including ETE, LNM (central and lateral), and TN. Al Afif et al. reported similar results for LNM (with folding rates for 3–9 foci and ≥10 foci) [49]. Tam et al. [18] reported significant positive correlations between TN and thyroid capsule invasion, ETE, and LNM in PTCs. In comparison, in this study, ROC curve analyses revealed that TN >1, a clinicopathological parameter indicating multifocal tumors with any TN, can predict LNM and recurrence. In addition, significant positive correlations were found between TN and the rates of LVI, ETE, and the need for RAI therapy in PTMCs. In general, the findings of this study on the relationship between multifocality and LNM are compatible with the literature, particularly in the context of PTMCs. An exception may be the findings on TN. On the other hand, the finding that the rate of ETE increased with the increase in TN in PTMCs was consistent with the results of previous studies. Additionally, the relationships between multifocality and TN with ETE and LVI in PTMCs were among the other noteworthy findings of this study.

The literature data on the effect of bilaterality on the prognosis of PTCs are controversial, as are the literature data on the effect of multifocality on the prognosis of PTCs [13,24,26,28,29,43,47,49,60,63,64,65,66]. Most studies found no relationship between LNM and the bilaterality of PTCs [26,43,47,60,65,66]. In contrast, Kim et al. [28] reported that ETE, LNM, advanced tumor stage, rate/dose of RAI therapy, and distant metastasis are more common in patients with bilateral tumors than in those with unilateral PTCs. Similarly, a few studies reported higher rates of bilateral tumors in older patients [28,29] with multifocal tumors [13,64] and tumors with follicular histology [13]. In parallel, the findings of this study on the relationships between the clinicopathological features of patients with unilateral unifocal PTCs, unilateral multifocal PTCs, or bilateral multifocal PTCs revealed higher recurrence rates in patients with bilateral multifocal tumors than in other patients. Multivariate analyses revealed bilaterality as a significant independent predictor for recurrence but not multifocality. An overview of this study’s findings on focality and laterality suggests that bilaterality rather than multifocality may be more predictive of recurrence in PTCs. Therefore, multifocality should not be overlooked in the pathology reports and clinical management of PTCs, especially PTMCs, as recommended in some current management guidelines [4,9].

The clonal nature of multifocal tumors has yet to be fully elucidated. Relevant data are limited to a few studies conducted with limited study groups. These studies reported that tumors with similar molecular characteristics may represent the same clone and the intraglandular spread of the primary tumor via the lymphatic network, which is also associated with LNM. On the other hand, tumors with different molecular characteristics may consist of different clones and, therefore, feature various molecular alterations and phenotypic features, e.g., different histological subtypes, bilaterality, and prognosis [50,67,68,69]. According to the first scenario, if multifocal PTCs represent an intraglandular metastasis via the lymphatic network, multifocality may indicate LNM [68,70]. As a matter of fact, in our previous study, we observed that intraglandular dissemination predicted LNM in multifocal tumors [70]. In line with Lin et al.’s findings, we found the rates of LVI and LNM to be higher in multifocal PTMCs than in unifocal PTMCs [68]. Additionally, we found a positive correlation between the rate of LVI and TN. All in all, these findings suggest that multifocal tumors are an intraglandular dissemination of the dominant tumor clone via the lymphatic network, and an increase in TN may resemble the increased circulation of neoplastic clones in the lymphatic network, increasing the likelihood of LNM. According to the second scenario, increased TN may result in increased heterogeneity in tumor clones and behavior. Accordingly, in patients with multifocal tumors, each tumor focus likely features independent tumors and independent clones that behave independently. Therefore, the characteristics of the largest tumor foci may not be sufficient to decide on the treatment of these tumors. An overview of this study’s findings and the literature data indicates the need for meticulous clinical management of these tumors, including a more careful evaluation of lymph nodes in multifocal PTMCs than in unifocal PTMCs, and suggests that multifocality may indicate the need for closer clinical follow-up of patients with PTCs, particularly PTMCs, regardless of the underlying scenario. Taking TN into account in the clinical management of these tumors may benefit the clinical course of PTMCs.

The debate about the clonal origin of multifocal tumors can also be considered in terms of the bilaterality of the tumor. As a reason, tumors with different molecular alterations may exhibit various phenotypic features such as bilaterality. Additionally, intraglandular circulation via the lymphatic network via the isthmus can result in the bilateral presentation of tumors with similar tumor clones [50,67,68,69]. In both cases, bilaterality may reflect higher tumor traffic in the intraglandular circulation or higher heterogeneity of tumors than multifocality. Therefore, careful risk stratification and close clinical follow-up would be appropriate in the case of bilateral tumors. However, in order to fully elucidate this debate, molecular analyses on the origin of bilateral tumors are needed within the scope of a larger case series.

In the last decade, novel parameters, including TTD [62], total surface area (TSA) [25], and tumor diameter ratio (TDR) [16], have been introduced as predictors of PTC prognosis. Zhao et al. [26] investigated the effect of TTD on central LNM in PTMCs. They reported a higher rate of LNM in patients with multifocal PTMCs with a TTD of >10 mm than in those with unifocal PTMCs. In contrast, Pyo et al. [25] reported that the TTD cutoff value of 10 mm is not a significant predictor of LNM in multifocal PTMCs and suggested using TSA instead. In a more recent study, Tam et al. [18] reported that patients with multifocal PTMCs with a TTD of >10 mm had higher rates of ETE, LNM, and capsular invasion than those with unifocal PTMCs. In addition, they stated that they did not find any significant difference between patients with multifocal PTMCs with a TTD of >10 mm and those with unifocal PTCs with a PTD of >10 mm in any of the parameters they investigated. Another study [32] reported higher rates of thyroid capsule invasion, LNM, and TN in patients with multifocal pT1a tumors with a TTD of 1–2 cm, suggesting that multifocal pT1a tumors with a TTD of 1–2 cm should be managed as pT1b tumors. In parallel with Tam et al.’s study [18], Liu et al. reported higher rates of LNM, ETE, and local infiltration in patients with multifocal PTMCs with a TTD higher than 10 mm and equal to or lower than 20 mm [71]. On the other hand, Xue et al. [72] reported that TTD >10 mm is a risk factor for the recurrence of unilateral multifocal PTMCs in patients with hemithyroidectomy. Along these lines, Wu et al. [62] reported that TTD is a more appropriate parameter than PTD for predicting LNM in unilateral multifocal PTCs. Based on the findings of the previous studies [18,25,26,71], 10 mm and 20 mm were taken as the cutoff values for TTD when investigating the relationships between clinicopathological features and TTD. Consequently, it was found that TTDs larger than 10 mm at the least or 20 mm were associated with larger PTDs (>10 mm); the presence of LVI, LNM, and ETE; and the need for RAI therapy. On the other hand, while TTD >10 mm was found to be significantly associated with the *BRAFV600E* mutation, TTD >20 mm was not. The recurrence rates for PTCs with a TTD of >20 mm were significantly higher than PTCs with a TTD of ≤20 mm. In addition, recurrence was more common, albeit not significantly, in PTCs with a TTD of >10 mm. PTCs with a TTD of >10 mm and a TTD of >20 mm featured PNI more frequently than PTCs with a TTD of ≤10 mm and a TTD of ≤20 mm. These findings are compatible with the literature data on the effect of TTD on tumor behavior in PTCs. The ROC curve analysis of TTD’s prognostic value in predicting LNM and recurrence revealed that patients with PTCs with a TTD of >11.5 mm may be more likely to have LNM, while patients with PTCs with a TTD of >19.5 mm may be more likely to have recurrence. The comparison of the clinicopathological features between patients with unifocal PTCs with a PTD of >10 mm and those with multifocal PTMCs with a TTD of >10 mm and a TTD of >20 mm did not reveal any significant difference, except for higher rates of LVI and the need for RAI therapy in patients with unifocal PTCs with a PTD of >10 mm, in line with the findings reported by Tam et al. [18], indicating that multifocal PTMCs with a TTD larger than at least 10 mm may show mostly similar phenotypic and predictive characteristics with unifocal PTCs with a PTD of >10 mm. Hence, increased TTD may reflect the increasing tumor burden in PTCs. Additionally, TTD may be considered the numerical evidence of tumor dissemination. A Consensus Report of the European Society of Endocrine Surgeons [41] recommended prophylactic central lymph node dissection for PTCs with a TTD of >10 mm and completion surgery in cases with high TN and saw no detriment to completion surgery and/or RAI therapy for multifocal PTCs diagnosed via hemithyroidectomy. In sum, TTD may be a parameter with more prognostic value for the clinical management of PTCs, especially multifocal PTMCs. Additionally, as Tam et al. [32] noted, >10 mm may be a better TTD cutoff value for PTMCs than >20 mm.

A thorough review of the literature revealed a limited number of studies on the impact of TTD on PTC prognosis since Buffet et al. [73] introduced the term TTD in 2012 (Table 6). Most of these studies used >10 mm as the TTD cutoff value. The sample size in these studies ranged between 82 and 2273 patients. Half of these studies featured a follow-up period, ranging between approximately 3.5 and 6.5 years, except for one study, which featured a follow-up period of 10 years. Some of these studies investigated the effect of TTD on LNM, while others explored the effect of TTD on recurrence. Only one study reported the impact of TTD on both LNM and recurrence. Almost all of these studies evaluating the effect of TTD on LNM and/or recurrence reported that TTD is significantly associated with recurrence and/or LNM. In comparison, the findings of this study investigating the impact of TTD on both LNM and recurrence on 706 patients for a median follow-up period of 9.3 (min 6.8, max. 20) years revealed that TTD ≥ 10 mm may significantly predict LNM and recurrence in PTCs. Nevertheless, large case series with more extended follow-up periods and optimized methodologies are needed to shed more light on the efficacy of TTD as a prognostic factor predicting LNM and recurrence in PTC.

Finally, we evaluated the parameters significantly predicting LNM and recurrence in PTCs. The univariate analyses revealed that TTD > 11.5 mm, TTD > 10 mm, and PNI had significant prognostic values in predicting LNM, whereas PNI, TTD > 20 mm, TTD > 19.5 mm, TTD > 10 mm, and bilaterality, along with other factors included in the current recurrence risk categorization models, had significant prognostic values in predicting recurrence (Table 4). Further analysis of these factors with multivariate analysis revealed that LVI and ETE are independent predictors of LNM, whereas LVI and TTD > 10 mm (17,912 (6,791–47,239)) are independent predictors of recurrence (Table 5). The results of these logistic regression analyses were compatible with current management guidelines [4]. The most notable of the univariate analysis findings are that PNI and TTD > 10 mm parameters are significant risk factors for LNM, and bilaterality, PNI, and TTD > 10 mm parameters are significant risk factors for recurrence. As discussed before, bilaterality and TTD may reflect a higher tumor burden. In conclusion, the use of TTD, which includes quantitative data on tumor volume, particularly the TTD cutoff value of >10 mm, as a prognostic marker may represent a more accurate approach in the clinical management of multifocal PTCs, especially PTMCs.

Perineural invasion, which may present in a clinically aggressive manner in many carcinomas, including breast and pancreatic carcinomas, involves a distinct metastatic pathway [79,80]. Although PNI is very rare in PTCs (1.5–1.8%) [80,81], some recent studies reported that increased nerve density and perineural invasion may be predictors of poor prognosis in terms of LVI, ETE, LNM, and extranodal extension (ENE) in PTCs [79,80,81]. Limberg et al. [80] reported higher rates of ENE in intrathyroidal tumors with PNI than those without PNI. Rowe et al. [79] reported a significant relationship between increased nerve density and ETE in PTCs. They mentioned the relationship between nerve density and the neurotrophic pro-nerve growth factor (proNGF) expression by cancer cells, possibly playing a role in increasing nerves in the tumor microenvironment. Sezer et al. [81] reported a 10 times higher PNI rate among patients with LVI. In comparison, in this study, the rate of PNI was found to be 1.8% (13 of 706 patients), in line with the findings of previous studies [80,81]. Univariate analysis revealed the presence of PNI as a significant risk factor for LNM and recurrence. In addition, ETE was present in all patients with PNI. The *BRAFV600E* mutation and LVI were present in 4 and 7 of the 13 patients with PNI, respectively. Six patients had LNM, whereas the only one patient with distant metastasis had PNI. Hence, PNI may involve a distinct pathway in relation to the local extension and metastatic process of PTCs. Lymphatic metastasis via the perineural space may be another possible scenario. However, further studies on the tumor microenvironment are needed in order to clarify the effects and the role of PNI in the spread of PTCs.

There were some limitations to this study. The retrospective single-center design of the study constitutes its primary limitation. The relatively low number of cases may also be deemed a limitation. The fact that approximately 5% of the cases had undergone hemithyroidectomy may be deemed another limitation. Since central lymph node dissection was performed only in patients with radiological or clinical preoperative/intraoperative suspicion of metastasis, lymph node dissection was evaluated in only slightly more than half of the patients. Hence, the fact that not all patients underwent central lymph node dissection may be deemed another limitation as it would affect the lymph node status of the patients. The fact that not all patients underwent *BRAFV600E* mutation analysis and only the molecular data of the dominant tumor were available may be deemed another limitation as they would affect the evaluations of molecular analyses. Lastly, given that this study involved the retrospective evaluation of pathology reports, a distinction between microscopic ETE and gross ETE could not be achieved. On the other hand, the assessment of all patients by a multidisciplinary council and a relatively more extended median follow-up period of 9.3 years may be considered the strengths of the study.

## 5. Conclusions

In conclusion, bilaterality and multifocality may impact tumor behavior in PTCs, particularly in PTMCs. Multifocal PTMCs with a TTD of >10 mm and unifocal PTCs with a PTD of >10 mm may have similar risk factors for tumor recurrence. TTD >10 mm may be a significant risk factor for recurrence and LNM. In addition to TTD and other risk factors included in recurrence risk categorization, PNI may be neglected as an indicator of LNM and recurrence in PTCs. Therefore, considering TTD, bilaterality, multifocality, and PNI in recurrence risk categorization models and adopting a clinical approach that takes into account multifocal PTMCs with TTD >10 mm along with unifocal PTCs with PTD >10 mm may be more useful in terms of the clinical management of the disease. Further large case series with more extended follow-up periods and meta-analyses may provide more data on the impact of focality, laterality, and TTD on the prognosis of PTCs.

## Figures and Tables

**Table 1:** Clinicopathological characteristics of the patients with papillary thyroid carcinomas and papillary microcarcinomas.

		In Papillary Carcinomas *n* (%)	Total	In Papillary Microcarcinomas *n* (%)	Total
**Age**	**≤45 years**	235 (33.3)	706	123 (30.3)	406
	**>45 years**	471 (66.7)		283 (69.7)	
**Gender**	**Female**	589 (83.4)	706	351 (86.5)	406
	**Male**	117 (16.6)		55 (13.5)	
**Surgery Procedure**	**Total thyroidectomy**	668 (94.6)	706	372 (91.6)	406
	**Lobectomy**	38 (5.4)		34 (8.4)	
	**+ LN dissection**	434 (61.5)		236 (58.1)	
**Lymphocytic thyroiditis**	**Absent**	411 (58.3)	706	228 (56.2)	406
	**Present**	295 (41.7)		178 (43.8)	
**Primary tumor diameter**	**≤10 mm**	406 (57.5)	706	406 (100.0)	406
	**>10 mm**	300 (42.5)			
**Total tumor** **diameter: 10 mm**	**≤10 mm**	323 (45.2)	706	316 (77.8)	406
	**>10 mm**	383 (54.2)		90 (22.2)	
**Total tumor** **diameter: 20 mm**	**≤20 mm**	528 (74.8)	706	398 (98.0)	406
	**>20 mm**	178 (25.2)		8 (2.0)	
**Histological** **subtype**	**CPTC**	346 (49.0)	706	210 (51.7)	406
	**IFPTC**	336 (47.6)		188 (46.3)	
	**ASPTC**	24 (3.4)		8 (2.0)	
**Tumor laterality**	**Unilateral**	440 (65.9)	668	269 (72.3)	372
	**Bilateral**	228 (34.1)		103 (27.7)	
**Tumor focality**	**Unifocal**	340 (48.2)	706	219 (53.9)	406
	**Multifocal**	366 (51.8)		187 (46.1)	
**Tumor laterality** **and focality**	**Unilateral/unifocal**	308 (46.1)	668	190 (51.1)	372
	**Unilateral/multifocal**	134 (20.1)		80 (21.5)	
	**Bilateral/multifocal**	226 (33.8)		102 (27.4)	
**Tumor foci** **(4-tiered system)**	**1**	342 (48.4)	706	219 (54.0)	406
	**2**	183 (25.9)		104 (25.6)	
	**3**	79 (11.2)		42 (10.3)	
	**4+**	102 (14.5)		41 (10.1)	
**Tumor foci** **(3-tiered system)**	**1**	342 (48.4)		219 (54.0))	
	**2**	183 (25.9)		104 (25.6)	
	**3+**	181 (25.7)		83 (20.4)	
**Lymphovascular invasion**	**Absent**	639 (90.5)	706	393 (96.8)	406
	**Present**	67 (9.5)		13 (3.2)	
**Perineural** **invasion**	**Absent**	693 (98.2)	706	403 (99.3)	406
	**Present**	13 (1.8)		3 (0.7)	
**Lymph node metastasis**	**Absent**	362 (83.4)	434	210 (89.0)	236
	**Present**	72 (16.6)		26 (11.0)	
**Extrathyroidal extension**	**Absent**	566 (80.2)	706	357 (87.9)	406
	**Present**	140 (19.8)		49 (12.1)	
**Radioactive** **iodine therapy**	**Absent**	326 (46.2)	706	319 (78.6)	406
	**Present**	380 (53.8)		87 (21.4)	
**Recurrence**	**Absent**	684 (96.9)	706	399 (98.3)	406
	**Present**	22 (3.1)		7 (1.7)	
**Distant** **metastasis**	**Absent**	705 (99.9)	706	406 (100.0)	406
	**Present**	1 (0.1)		-	
**BRAFV600E**	**Wild-type**	362 (79.6)	455	218 (84.2)	259
	**Mutated**	93 (20.4)		41 (15.8)	

LN: lymph node, CPTC: classic papillary thyroid carcinoma, IFPTC: infiltrative follicular subtype papillary thyroid carcinoma, ASPTC: aggressive subtype papillary thyroid carcinoma, + LN dissection: central or/and cervical lymph node dissection.

**Table 2:** Comparisons of clinicopathological features by “total tumor diameter”.

Variables		TTD ≤10 mm *n*: 323	TTD >10 mm *n*: 383	*p*	TTD ≤20 mm *n*: 528	TTD >20 mm *n*: 178	*p*
**Age**	**≤45 years**	102 (43.4)	133 (56.6)	0.377	172 (73.2)	63 (26.8)	0.490
	**>45 years**	221 (46.9)	250 (53.1)		356 (75.6)	115 (24.4)	
**Gender**	**Female**	280 (47.5)	309 (52.5)	**0.032**	451 (76.6)	138 (23.4)	**0.014**
	**Male**	43 (36.8)	74 (63.2)		77 (65.8)	40 (34.2)	
**Lymphocytic thyroiditis**	**Absent**	191 (46.5)	220 (53.5)	0.675	307 (74.7)	104 (25.3)	0.932
	**Present**	132 (44.7)	163 (55.3)		221 (74.9)	74 (25.1)	
**Primary tumor diameter**	**≤10 mm**	316 (77.8)	90 (22.2)	**<0.001**	398 (98.0)	8 (2.0)	**<0.001**
	**>10 mm**	7 (2.3)	293 (97.7)		130 (43.3)	170 (56.7)	
**Histological** **subtype**	**CPTC**	164 (47.4)	182 (52.6)	0.091	263 (76.0)	83 (24.0)	**0.002**
	**IFPTC**	153 (45.5)	183 (54.5)		255 (75.9)	81 (24.1)	
	**ASPTC**	6 (25.0)	18 (75.0)		10 (41.7)	14 (58.3)	
**LVI**	**Absent**	317 (49.6)	322 (50.4)	**<0.001**	493 (77.1)	146 (22.9)	**<0.001**
	**Present**	6 (9.0)	61 (91.0)		35 (52.2)	32 (47.8)	
**PNI**	**Absent**	320 (46.2)	373 (53.8)	**0.008**	523 (75.5)	170 (24.5)	**0.017**
	**Present**	3 (23.1)	10 (76.9)		5 (38.5)	8 (61.5)	
**LNM**	**Absent**	161 (44.5)	201 (55.5)	**<0.001**	268 (74.0)	94 (26.0)	**0.044**
	**Present**	16 (22.2)	56 (77.8)		47 (65.3)	25 (34.7)	
**ETE**	**Absent**	293 (51.8)	273 (48.2)	**<0.001**	441 (77.9)	125 (22.1)	**<0.001**
	**Present**	30 (21.4)	110 (78.5)		87 (62.1)	53 (37.9)	
**RAI therapy**	**Absent**	266 (81.6)	60 (18.4)	**<0.001**	320 (98.2)	6 (1.8)	**<0.001**
	**Present**	57 (15.0)	323 (85.0)		208 (54.7)	172 (45.3)	
**Recurrence**	**Absent**	318 (46.5)	371 (53.5)	0.078	522 (75.5)	169 (24.5)	**0.005**
	**Present**	5 (22.7)	17 (77.3)		10 (40.0)	12 (60.0)	
**Distant metastasis**	**Absent**	323 (45.8)	382 (54.2)	0.542	528 (74.9)	177 (25.1)	0.252
	**Present**	0 (0.0)	1 (100.0)		0 (0.0)	1 (100.0)	
**BRAFV600E**	**Wild-type**	173 (47.8)	189 (52.2)	**0.012**	271 (74.9)	91 (25.1)	0.238
	**Mutated**	31 (33.3)	62 (66.7)		64 (68.8)	29 (31.2)	

TTD: total tumor diameter, CPTC: classic papillary thyroid carcinoma, IFPTC: infiltrative follicular subtype papillary thyroid carcinoma, ASPTC: aggressive subtype papillary thyroid carcinoma, LVI: lymphovascular invasion, PNI: perineural invasion, LNM: lymph node metastasis, ETE: extrathyroidal extension.

**Table 3:** Comparisons of clinicopathological features between unifocal papillary thyroid carcinomas sized larger than 10 mm and multifocal papillary microcarcinomas with total tumor diameters >10 mm, >20 mm.

Variables		PTD >10 mm Unifocal *n* (%)	PTD ≤10 mm TTD >10 mm Multifocal *n* (%)	*p*	PTD >10 mm Unifocal *n* (%)	PTD ≤10 mm TTD >20 mm Multifocal *n* (%)	*p*
**Age**	**≤45 years**	53 (69.7)	23 (30.3)	**0.011**	53 (96.4)	2 (3.6)	0.284
	**>45 years**	68 (50.7)	66 (49.3)		68 (91.9)	6 (8.1)	
**Gender**	**Female**	94 (55.0)	77 (45.0)	0.148	94 (93.1)	7 (6.9)	1.000
	**Male**	27 (69.2)	12 (30.8)		27 (96.4)	1 (3.6)	
**Histological** **subtype**	**CPTC**	47 (50.0)	47 (50.0)	0.130	47 (87.0)	7 (13.0)	**0.020**
	**IFVPTC**	71 (64.0)	40 (36.0)		71 (98.6)	1 (1.4)	
	**ASPTC**	3 (60.0)	2 (40.0)		3 (100.0)	0 (0.0)	
**LVI**	**Absent**	95 (54.0)	81 (46.0)	**0.025**	95 (93.1)	7 (6.9)	0.522
	**Present**	26 (76.5)	8 (23.5)		26 (96.3)	1 (3.7)	
**PNI**	**Absent**	115 (56.7)	88 (43.3)	0.243	115 (93.5)	8 (6.5)	1.000
	**Present**	6 (85.5)	1 (14.3)		6 (100.0)	0 (0.0)	
**LNM**	**Absent**	59 (52.7)	53 (47.3)	0.136	59 (90.8)	6 (9.2)	1.000
	**Present**	20 (66.7)	10 (33.3)		20 (95.2)	1 (4.8)	
**ETE**	**Absent**	76 (52.8)	68 (47.2)	0.052	76 (93.8)	5 (6.2)	1.000
	**Present**	45 (68.2)	21 (31.8)		45 (93.8)	3 (6.3)	
**Radioactive** **iodine therapy**	**Absent**	4 (7.1)	52 (92.9)	**<0.001**	4 (57.1)	3 (42.9)	**0.005**
	**Present**	117 (76.0)	37 (24.0)		117 (95.9)	5 (4.1)	
**Recurrence**	**Absent**	117 (57.4)	87 (42.6)	0.264	117 (93.6)	8 (6.4)	1.000
	**Present**	4 (66.7)	2 (33.3)		4 (100.0)	0 (0.0)	
**BRAFV600E**	**Wild-type**	48 (51.6)	45 (48.4)	0.128	48 (92.3)	4 (7.7)	1.000
	**Mutated**	24 (68.6)	11 (31.4)		24 (92.3)	2 (7.7)	

PTD: primary tumor diameter, TTD: total tumor diameter, CPTC: classic papillary thyroid carcinoma, IFPTC: infiltrative follicular subtype papillary thyroid carcinoma, ASPTC: aggressive subtype papillary thyroid carcinoma, LVI: lymphovascular invasion, PNI: perineural invasion, LNM: lymph node metastasis, ETE: extrathyroidal extension.

**Table 4:** Effects of clinicopathological features on lymph node metastasis and recurrence.

Univariate Analyses											
Variables		Lymph Node Metastasis		Univariate		Variables		Recurrence		Univariate	
		Absent	Present	*p*	OR (95% CI)			Absent	Present	*p*	OR (95% CI)
**Age**	**≤45 years**	120 (77.9)	34 (22.1)	**0.018**	0.537 (0.321–0.899)	**Age**	**≤45 years**	225 (95.7)	10 (4.3)	0.223	0.588 (0.250–1.382)
	**>45 years**	243 (86.8)	37 (13.2)				**>45 years**	459 (97.5)	12 (2.5)		
**Gender**	**Female**	321 (87.2)	47 (12.8)	**<0.001**	3.903 (2.169–7.023)	**Gender**	**Female**	572 (97.1)	17 (2.9)	0.433	1.502 (0.543–4.155)
	**Male**	42 (63.6)	24 (36.4)				**Male**	112 (95.7)	5 (4.3)		
**Lymphocytic thyroiditis**	**Absent**	184 (81.8)	41 (18.2)	0.277	0.752 (0.450–1.257)	**Lymphocytic thyroiditis**	**Absent**	396 (96.4)	15 (3.6)	0.339	0.642 (0.258–1.594)
	**Present**	179 (85.6)	30 (14.4)				**Present**	288 (97.6)	7 (2.4)		
**Tumor focality** and laterality *	**Unilateral Unifocal**	159 (84.6)	29 (15.4)			**Tumor focality** and laterality **	**Unilateral Unifocal**	302 (97.7)	7 (2.3)		
	**Unilateral Multifocal**	66 (82.5)	14 (17.5)	0.672	1.163 (0.578–2.341)		**Unilateral Multifocal**	133 (97.8)	3 (2.2)	0.969	0.973 (0.248–3.821)
	**Bilateral Multifocal**	126 (81.8)	28 (18.2)	0.497	1.218 (0.689–2.153)		**Bilateral Multifocal**	214 (94.7)	12 (5.3)	0.068	2.419 (0.937–6.246)
**Tumor focality**	**Unifocal**	169 (85.4)	29 (14.6)	0.378	1.262 (0.753–2.114)	**Tumor focality**	**Unifocal**	333 (97.9)	7 (2.1)	0.126	2.033 (0.819–5.048)
	**Multifocal**	194 (82.2)	42 (17.8)				**Multifocal**	351 (95.9)	15 (4.1)		
**Primary tumor diameter**	**≤1 cm**	211 (89.4)	25 (10.6)	**0.001**	2.554 (1.504–4.338)	**Primary tumor diameter**	**≤1 cm**	399 (98.3)	7 (1.7)	**0.018**	3.000 (1.208–7.452)
	**>1 cm**	152 (76.8)	46 (23.2)				**>1 cm**	285 (95.0)	15 (5.0)		
**Tumor laterality**	**Unilateral**	223 (83.8)	43 (16.2)	0.637	1.134 (0.672–1.914)	Tumor laterality *	**Unilateral**	433 (97.7)	10 (2.3)	**0.044**	2.406 (1.023–5.656)
	**Bilateral**	128 (82.1)	28 (17.9)				**Bilateral**	216 (94.7)	12 (5.3)		
**Total tumor** **diameter: 10 mm**	**≤10 mm**	162 (91.5)	15 (8.5)	**<0.001**	3.009 (1.641–5.517)	**Total tumor** **diameter: 10 mm**	**≤10 mm**	318 (98.5)	5 (1.5)	**0.035**	2.954 (1.078–8.098)
	**>10 mm**	201 (78.2)	56 (21.8)				**>10 mm**	366 (95.6)	17 (4.4)		
**Total tumor** **diameter: 20 mm**	**≤20 mm**	269 (85.4)	46 (14.6)	0.109	1.555 (0.906–2.671)	**Total tumor** **Diameter: 20 mm**	**≤20 mm**	518 (98.1)	10 (1.9)	**0.003**	3.745 (1.589–8.824)
	**>20 mm**	94 (79.0)	25 (21.0)				**>20 mm**	166 (93.3)	12 (6.7)		
**Total tumor** **diameter: 11.5 mm**	**≤11.5 mm**	176 (91.7)	17 (8.3)	**<0.001**	3.235 (1.787–5.857)	**Total tumor** **diameter: 19.5 mm**	**≤19.5 mm**	345 (98.6)	5 (1.4)	**0.016**	3.460 (1.262–9.484)
	**>11.5 mm**	187 (77.3)	55 (22.7)				**>19.5 mm**	339 (95.2)	17 (4.8)		
**Histological** **subtype**	**CPTC**	183 (76.9)	55 (23.1)	**<0.001**	0.261 (0.137–0.494)	**Histological** **subtype**	**CPTC**	333 (96.2)	13 (3.8)	0.126	0.466 (0.175–1.240)
	**IFPTC**	166 (92.7)	13 (7.3)				**IFVPTC**	330 (98.2)	6 (1.8)		
	**ASPTC**	14 (82.4)	3 (17.6)				**ASPTC**	21 (87.5)	3 (12.5)		
**LVI**	**Absent**	347 (92.0)	30 (8.0)	**<0.001**	29.640 (14.901–58.956)	**LVI**	**Absent**	626 (98.0)	13 (2.0)	**<0.001**	7.472 (3.064–18.222)
	**Present**	16 (28.1)	41 (71.9)				**Present**	58 (86.6)	9 (13.4)		
**PNI**	**Absent**	359 (84.7)	65 (15.3)	**<0.001**	8.285 (2.275–30.170)	**PNI**	**Absent**	673 (97.1)	20 (2.9)	**0.024**	6.118 (1.272–29.434)
	**Present**	4 (40.0)	6 (60.0)				**Present**	11 (84.6)	2 (15.4)		
**ETE**	**Absent**	302 (91.2)	29 (8.8)	**<0.001**	7.170 (4.148–12.395)	**ETE**	**Absent**	555 (98.1)	11 (1.9)	**0.001**	4.302 (1.825–10.141)
	**Present**	61 (59.2)	42 (40.8)				**Present**	129 (92.1)	11 (7.9)		
***BRAFV600E***	**Wild-type**	190 (87.2)	28 (12.8)	**0.001**	2.850 (1.494–5.437)	***BRAFV600E***	**Wild-type**	354 (97.8)	8 (2.2)	**0.016**	3.602 (1.271–10.204)
	**Mutated**	50 (70.4)	21 (29.6)				**Mutated**	86 (92.5)	7 (7.5)		

CPTC: classic papillary thyroid carcinoma, IFPTC: infiltrative follicular subtype papillary thyroid carcinoma, ASPTC: aggressive subtype papillary thyroid carcinoma, LVI: lymphovascular invasion, PNI: perineural invasion, ETE: extrathyroidal extension. * Including 422 patients with total thyroidectomy and lymph node dissection ** Including 668 patients with total thyroidectomy and 3 patients with surgery for recurrence.

**Table 5:** Significant clinicopathological factors for lymph node metastasis and recurrence.

Multivariate Analyses					
Lymph Node Metastasis			Recurrence		
Variables	*p*	OR (95% CI)	Variables	*p*	OR (95% CI)
**Lymphovascular invasion**	**0.001**	10.305 (2.591–40.987)	**Total tumor diameter: 10 mm**	**<0.001**	17.912 (6.791–47.239)
**Extrathyroidal extension**	**0.013**	4.608 (1.388–15.302)	**Lymphovascular invasion**	**0.004**	12.146 (2.269–65.019)

**Table 6:** Review of the previous studies investigating the effect of total tumor diameter on lymph node metastasis and recurrence.

Study	Year	Country	Study Group	Number of Patients Included in the Study	Suggested Cutoff Value for TTD	Follow-Up Period	Lymph Node Metastasis	Recurrence
Buffet et al. [73]	2012	France	PTMC	1669	>20 mm	4.7 years (1 month–37.9 years).	NE	Significantly associated
Zhao et al. [26]	2013	China	PTMC	212	>10 mm	Lacking	Significantly associated	NE
Pyo et al. [25]	2015	Korea	PTMC	384	>10 mm	Lacking	Not associated	NE
Chereau et al. [74]	2016	Germany	PTC/PTMC	2273	Not suggested	6.5 years (1–36.4 years)	NE	Significantly associated
Tam et al. [18]	2016	Turkey	PTC/PTMC	912	>10 mm	37 months (6–99 months)	Significantly associated	NE
Wang et al. [42]	2017	China	PTC	1084	Not suggested	Lacking	Significantly associated	NE
Xue et al. [72]	2017	China	PTMC	97	>10 mm	>10 years for 89 patients	NE	Significantly associated
Feng et al. [34]	2020	China	PTC	442	>10 mm	43 months (11–99 months)	Significantly associated	Significantly associated
Manso et al. [75]	2020	Italy	PTC	370	≥40 mm	69 months (42–92 months)	Not reported	Significantly associated
Hitu et al. [76]	2021	Romania	PTMC	82	>10 mm	Lacking	Significantly associated	NE
Jiang et al. [77]	2022	China	PTMC	560	>10 mm	Lacking	Significantly associated	NE
Kwon et al. [78]	2022	Korea	PTC	1288	Not suggested	6.4 years (4.4–8.7 years)	Not reported	Significantly associated
Wu et al. [62]	2023	China	PTC	1936	Not suggested	Not reported	Significantly associated	Not reported
The present study; Can et al.	2023	Turkey	PTC/PTMC	706	>10 mm	112 months (82–240 months)	Significantly associated	Significantly associated

TTD: total tumor diameter, PTMC: papillary microcarcinoma PTC: papillary thyroid carcinoma, NE: not evaluated.

## Data Availability

The data presented in this study are available on request from the corresponding author.

## Supplementary Materials

The following supporting information can be downloaded at https://www.mdpi.com/article/10.3390/diagnostics14030272/s1: Table S1: Comparisons of clinicopathological features by “tumor focality” in all papillary thyroid carcinomas and papillary microcarcinomas. Table S2: Distribution of clinicopathological features by “tumor laterality” in the overall study group and patients with papillary microcarcinomas. Table S3: Distribution of clinicopathological features by “number of tumor foci”.
