# Supplementary material for: The Impact of Total Tumor Diameter on Lymph Node Metastasis and Tumor Recurrence in Papillary Thyroid Carcinomas
Source: Diagnostics (Basel). 2024 Jan 26;14(3):272. doi: 10.3390/diagnostics14030272 (PMC10854897; doi:10.3390/diagnostics14030272)
Supplement: Supplementary file 1 [file diagnostics-14-00272-s001.zip › diagnostics-2738486-supplementary.pdf]

**Supplemental Table S1.** Comparisons of clinicopathological features by "tumor focality" in all papillary thyroid carcinomas and papillary microcarcinomas.

| | | ***In all papillary thyroid carcinomas n: 706*** | | | | ***In papillary microcarcinomas n: 406*** | | | |
|---|---|---|---|---|---|---|---|---|---|
| ***Variables*** | | **Unifocal n: 340 n (%)** | **Multifocal n: 366 n (%)** | **Total n** | **p** | **Unifocal n: 219 n (%)** | **Multifocal n: 187 n (%)** | **Total n** | **p** |
| ***Age*** | **≤45 years** | 128 (54.5) | 107 (45.5) | 235 | **0.018** | 75 (61.0) | 48 (39.0) | 123 | 0.061 |
| | **>45 years** | 212 (45.0) | 259 (55.0) | 471 | | 144 (50.9) | 139 (49.1) | 283 | |
| ***Gender*** | **Female** | 289 (49.1) | 300 (50.9) | 589 | 0.279 | 195 (55.6) | 156 (44.4) | 351 | 0.067 |
| | **Male** | 51 (43.6) | 66 (56.4) | 117 | | 24 (43.6) | 31 (56.4) | 55 | |
| ***Lymphocytic thyroiditis*** | **Absent** | 207 (50.5) | 204 (49.5) | 411 | 0.176 | 129 (56.6) | 99 (43.4) | 228 | 0.243 |
| | **Present** | 133 (45.1) | 162 (54.9) | 295 | | 90 (50.6) | 88 (49.4) | 178 | |
| ***Primary tumor diameter*** | **≤10 mm** | 219 (53.9) | 187 (46.1) | 406 | **<0.001** | 219 (53.9) | 187 (46.1) | 406 | * |
| | **>10 mm** | 121 (40.3) | 179 (59.7) | 300 | | | | | |
| ***Histological subtype*** | **CPTC** | 160 (46.2) | 186 (53.8) | 346 | 0.065 | 113 (53.8) | 97 (46.2) | 210 | 0.971 |
| | **IFPTC** | 173 (51.5) | 163 (48.5) | 336 | | 102 (54.3) | 86 (45.7) | 188 | |
| | **ASPTC** | 7 (29.2) | 17 (70.8) | 24 | | 4 (50.0) | 4 (50.0) | 8 | |
| ***LVI*** | **Absent** | 311 (48.7) | 328 (51.3) | 639 | 0.401 | 216 (55.0) | 177 (45.0) | 393 | **0.023** |
| | **Present** | 29 (43.3) | 38 (56.7) | 67 | | 3 (23.1) | 10 (76.9) | 13 | |
| ***PNI*** | **Absent** | 333 (48.0) | 360 (52.0) | 693 | 0.893 | 218 (54.1) | 185 (45.9) | 403 | 0.441 |
| | **Present** | 7 (53.8) | 6 (46.2) | 13 | | 1 (33.3) | 2 (66.7) | 3 | |
| ***LNM*** | **Absent** | 169 (46.7) | 193 (53.3) | 362 | 0.291 | 110 (52.4) | 100 (47.6) | 210 | 0.081 |
| | **Present** | 29 (40.3) | 43 (59.7) | 72 | | 9 (34.7) | 17 (65.3) | 26 | |
| ***ETE*** | **Absent** | 277 (48.9) | 289 (51.1) | 566 | 0.404 | 201 (56.3) | 156 (43.7) | 357 | **0.008** |
| | **Present** | 63 (45.0) | 77 (55.0) | 140 | | 18 (36.7) | 31 (63.3) | 49 | |
| ***Radioactive iodine therapy*** | **Absent** | 193 (59.2) | 133 (40.8) | 326 | **<0.001** | 189 (59.2) | 130 (40.8) | 319 | **<0.001** |
| | **Present** | 147 (38.7) | 233 (61.3) | 380 | | 30 (34.5) | 57 (65.5) | 87 | |
| ***Recurrence*** | **Absent** | 336 (48.6) | 355 (51.4) | 691 | 0.155 | 218 (54.1) | 185 (45.9) | 403 | 0.441 |
| | **Present** | 7 (31.8) | 15 (68.2) | 22 | | 3 (42.9) | 4 (57.1) | 7 | |
| ***BRAFV600E*** | **Wild-type** | 167 (46.1) | 195 (53.9) | 362 | 0.448 | 119 (54.6) | 99 (45.4) | 218 | 0.994 |
| | **Mutated** | 47 (50.5) | 46 (49.5) | 93 | | 23 (56.1) | 18 (43.9) | 41 | |

**CPTC:** : Classic papillary thyroid carcinoma, **IFPTC:** Infiltrative follicular subtype papillary thyroid carcinoma, **ASPTC:** Aggressive subtype papillary thyroid carcinoma**, LVI:** Lymphovascular invasion, **PNI:** Perineural invasion**, LNM:** Lymph node metastasis, **ETE:** Extrathyroidal extension.

**Supplemental Table S2 . Comparisons of clinicopathological features by "number of tumor foci"**

***In whole study group.***

| ***Variables*** | | ***Number of tumor foci according to 4-tiered system n: 706*** | | | | | ***Number of tumor foci according to 3-tiered sytem n: 706*** | | | |
|---|---|---|---|---|---|---|---|---|---|---|
| | | **1 n (%)** | **2 n (%)** | **3 n (%)** | **4+ n (%)** | **p** | **1 n (%)** | **2 n (%)** | **3+ n (%)** | **p** |
| ***Age*** | **≤45 years** | 129 (54.9) | 58 (24.7) | 18 (7.7) | 30 (12.8) | **0.049** | 129 (54.9) | 58 (24.7) | 48 (20.4) | **0.031** |
| | **>45 years** | 213 (45.2) | 125 (26.5) | 61 (13.0) | 72 (15.3) | | 213 (45.2) | 125 (26.5) | 133 (28.2) | |
| ***Gender*** | **Female** | 291 (49.4) | 145 (24.6) | 66 (11.2) | 87 (14.8) | 0.352 | 291 (49.4) | 145 (24.6) | 153 (26.0) | 0.206 |
| | **Male** | 51 (43.6) | 38 (32.5) | 13 (11.1) | 15 (12.8) | | 51 (43.6) | 38 (32.5) | 28 (23.9) | |
| ***Lymphocytic thyroiditis*** | **Absent** | 209 (50.7) | 108 (26.3) | 41 (10.0) | 53 (12.9) | 0.254 | 209 (50.7) | 108 (26.3) | 94 (22.9) | 0.131 |
| | **Present** | 133 (45.1) | 75 (25.4) | 38 (12.9) | 49 (16.6) | | 133 (45.1) | 75 (25.4) | 87 (29.5) | |
| ***Primary tumor diameter*** | **≤10 mm** | 219 (35.9) | 104 (25.6) | 42 (10.3) | 41 (10.1) | **<0.001** | 219 (35.9) | 104 (25.6) | 83 (20.4) | **<0.001** |
| | **>10 mm** | 123 (41.0) | 79 (26.3) | 37 (12.3) | 61 (20.3) | | 123 (41.0) | 79 (26.3) | 98 (32.7) | |
| ***Histological subtype*** | **CPTC** | 162 (46.8) | 80 (23.1) | 38 (11.0) | 66 (19.1) | **<0.001** | 162 (46.8) | 80 (23.1) | 104 (30.1) | **0.006** |
| | **IFPTC** | 173 (51.5) | 91 (27.1) | 41 (12.2) | 31 (9.2) | | 173 (51.5) | 91 (27.1) | 72 (21.4) | |
| | **ASPTC** | 7 (29.2) | 12 (50.0) | 0 (0.0) | 5 (20.8) | | 7 (29.2) | 12 (50.0) | 5 (20.8) | |
| ***LVI*** | **Absent** | 313 (49.0) | 166 (26.0) | 73 (11.4) | 87 (13.6) | 0.264 | 313 (49.0) | 166 (26.0) | 160 (25.0) | 0.508 |
| | **Present** | 29 (43.3) | 17 (25.4) | 6 (9.0) | 15 (22.4) | | 29 (43.3) | 17 (25.4) | 21 (31.3) | |
| ***PNI*** | **Absent** | 335 (48.3) | 181 (26.1) | 78 (11.3) | 99 (14.3) | 0.688 | 335 (48.3) | 181 (26.1) | 177 (25.5) | 0.676 |
| | **Present** | 7 (53.8) | 2 (15.4) | 1 (7.7) | 3 (23.1) | | 7 (53.8) | 2 (15.4) | 4 (30.8) | |
| ***LNM*** | **Absent** | 170 (47.0) | 88 (24.3) | 42 (11.6) | 62 (17.1) | 0.658 | 170 (47.0) | 88 (24.3) | 104 (28.7) | 0.498 |
| | **Present** | 29 (40.3) | 23 (32.0) | 7 (9.7) | 13 (18.0) | | 29 (40.3) | 23 (32.0) | 20 (27.7) | |
| ***ETE*** | **Absent** | 279 (49.3) | 145 (25.6) | 64 (11.3) | 78 (13.8) | 0.696 | 279 (49.3) | 145 (25.6) | 142 (25.1) | 0.649 |
| | **Present** | 63 (45.0) | 38 (27.1) | 15 (10.7) | 24 (17.1) | | 63 (45.0) | 38 (27.1) | 39 (27.9) | |
| ***Radioactive iodine therapy*** | **Absent** | 193 (59.2) | 81 (24.8) | 29 (8.9) | 23 (7.1) | **<0.001** | 193 (59.2) | 81 (24.8) | 52 (16.0) | **<0.001** |
| | **Present** | 149 (39.2) | 102 (26.8) | 50 (13.2) | 79 (20.8) | | 149 (39.2) | 102 (26.8) | 129 (33.9) | |
| ***Recurrence*** | **Absent** | 338 (48.9) | 177 (25.6) | 78 (11.3) | 98 (14.2) | 0.209 | 338 (48.9) | 177 (25.6) | 176 (25.5) | 0.220 |
| | **Present** | 7 (31.8) | 8 (36.4) | 2 (9.1) | 5 (22.7) | | 7 (31.8) | 8 (36.4) | 7 (31.8) | |
| ***BRAFV600E*** | **Wild-type** | 167 (46.1) | 97 (26.8) | 42 (11.6) | 56 (15.5) | 0.685 | 167 (46.1) | 97 (26.8) | 98 (27.1) | 0.528 |
| | **Mutated** | 48 (51.6) | 20 (21.5) | 12 (12.9) | 13 (14.0) | | 48 (51.6) | 20 (21.5) | 25 (26.9) | |

**CPTC:** : Classic papillary thyroid carcinoma, **IFPTC:** Infiltrative follicular subtype papillary thyroid carcinoma, **ASPTC:** Aggressive subtype papillary thyroid carcinoma, **TTD:** Total tumor diameter**, LVI:** Lymphovascular invasion, **PNI:** Perineural invasion**, LNM:** Lymph node metastasis, **ETE:** Extrathyroidal extension.

**Supplemental Table S3.** Comparisons of clinicopathological features by tumor laterality and focality in papillary thyroid carcinomas and papillary microcarcinomas with total thyroidectomy.

| | | In whole study group with total thyroidectomy n: 668 | | | | | In papillary microcarcinomas with total thyroidectomy n: 372 | | | | |
|---|---|---|---|---|---|---|---|---|---|---|---|
| **Variables** | | **Unilateral/ Unifocal n (%)** | **Unilateral/ Multifocal n (%)** | **Bilateral/ Multifocal n (%)** | **Total n** | **p** | **Unilateral/ Unifocal n (%)** | **Unilateral/ Multifocal n (%)** | **Bilateral/ Multifocal n (%)** | **Total n** | **p** |
| **Age** | **≤45 years** | 117 (52.2) | 38 (17.0) | 69 (30.8) | 224 | 0.072 | 66 (57.9) | 21 (18.4) | 27 (23.7) | 114 | 0.217 |
| | **>45 years** | 191 (43.0) | 96 (21.6) | 157 (35.4) | 444 | | 124 (48.1) | 59 (22.9) | 75 (29.1) | 258 | |
| **Gender** | **Female** | 261 (46.9) | 107 (19.2) | 188 (33.8) | 556 | 0.449 | 169 (52.6) | 64 (19.9) | 88 (27.4) | 321 | 0.149 |
| | **Male** | 47 (42.0) | 27 (24.1) | 38 (33.9) | 112 | | 21 (41.2) | 16 (31.4) | 14 (27.5) | 51 | |
| **Lymphocytic thyroiditis** | **Absent** | 185 (47.9) | 77 (19.9) | 124 (32.1) | 386 | 0.484 | 110 (53.4) | 44 (21.4) | 52 (25.2) | 206 | 0.525 |
| | **Present** | 123 (43.6) | 57 (20.2) | 102 (36.2) | 282 | | 80 (48.2) | 36 (21.7) | 50 (30.1) | 166 | |
| **Primary tumor diameter** | **≤10 mm** | 190 (51.1) | 80 (21.5) | 102 (27.4) | 372 | **<0.001** | 190 (51.1) | 80 (21.5) | 102 (27.4) | 372 | * |
| | **>10 mm** | 118 (39.9) | 54 (18.2) | 124 (41.9) | 296 | | | | | | |
| **Histological subtype** | **CPTC** | 145 (44.1) | 65 (19.8) | 119 (36.2) | 329 | 0.102 | 99 (50.8) | 43 (22.1) | 53 (27.2) | 195 | 0.958 |
| | **IFPTC** | 156 (49.5) | 65 (20.6) | 94 (29.8) | 315 | | 87 (51.5) | 36 (21.3) | 46 (27.2) | 169 | |
| | **ASPTC** | 7 (29.2) | 4 (16.7) | 13 (54.2) | 24 | | 4 (50.0) | 1 (12.5) | 3 (37.5) | 8 | |
| **LVI** | **Absent** | 279 (46.4) | 124 (20.6) | 198 (32.9) | 601 | 0.286 | 187 (52.1) | 76 (21.2) | 96 (26.7) | 359 | 0.115 |
| | **Present** | 29 (46.1) | 10 (14.9) | 28 (41.8) | 67 | | 3 (23.1) | 4 (30.8) | 6 (46.2) | 13 | |
| **PNI** | **Absent** | 301 (46.0) | 131 (20.0) | 223 (34.0) | 655 | 0.710 | 189 (51.2) | 78 (21.1) | 102 (27.6) | 369 | 0.143 |
| | **Present** | 7 (53.8) | 3 (23.1) | 3 (23.1) | 13 | | 1 (33.3) | 2 (66.7) | 0 (0.0) | 3 | |
| **LNM** | **Absent** | 159 (45.4) | 64 (18.3) | 127 (36.3) | 350 | 0.659 | 110 (52.4) | 39 (18.6) | 61 (29.0) | 210 | 0.177 |
| | **Present** | 26 (39.4) | 13 (19.7) | 27 (40.9) | 66 | | 9 (34.6) | 8 (30.8) | 9 (34.6) | 26 | |
| **ETE** | **Absent** | 245 (46.4) | 107 (20.3) | 176 (33.3) | 528 | 0.867 | 172 (53.3) | 67 (20.7) | 84 (26.0) | 323 | 0.093 |
| | **Present** | 63 (45.0) | 27 (19.3) | 50 (35.7) | 140 | | 18 (36.7) | 13 (26.5) | 18 (36.7) | 49 | |

| | | | | | | | | | | | |
|---|---|---|---|---|---|---|---|---|---|---|---|
| **Radioactive iodine therapy** | **Absent** | 164 (56.2) | 54 (18.5) | 74 (25.3) | 292 | **<0.001** | 160 (56.1) | 53 (18.6) | 72 (25.3) | 285 | **0.002** |
| | **Present** | 144 (38.3) | 80 (21.3) | 152 (40.4) | 376 | | 30 (34.5) | 27 (31.0) | 30 (34.5) | 87 | |
| **Recurrence** | **Absent** | 304 (46.6) | 133 (20.4) | 216 (33.1) | 684 | **0.018** | 216 (58.5) | 83 (22.5) | 100 (27.0) | 369 | 0.812 |
| | **Present** | 7 (31.8) | 3 (13.6) | 12 (54.6) | 22 | | 3 (42.8) | 2 (28.6) | 2 (28.6) | 7 | |
| **BRAFV600E** | **Wild-type** | 148 (43.3) | 71 (20.8) | 123 (36.0) | 342 | 0.574 | 101 (50.5) | 41 (20.5) | 58 (29.0) | 200 | 0.904 |
| | **Mutated** | 45 (49.5) | 17 (18.7) | 29 (31.9) | 91 | | 21 (53.8) | 8 (20.5) | 10 (25.6) | 39 | |

**CPTC: :** Classic papillary thyroid carcinoma, **IFPTC:** Infiltrative follicular subtype papillary thyroid carcinoma, **ASPTC:** Aggressive subtype papillary thyroid carcinoma., **TTD:** Total tumor diameter**, LVI:** Lymphovascular invasion, **PNI:** Perineural invasion**, LNM:** Lymph node metastasis, **ETE:** Extrathyroidal extension.
